# Supplementary material for: Multi‐dataset identification of innovative feature genes and molecular mechanisms in keratoconus
Source: J Cell Mol Med. 2024 Sep 19;28(18):e70079. doi: 10.1111/jcmm.70079 (PMC11412914; doi:10.1111/jcmm.70079)
Supplement: Supplementary file 2 — Material S2. [file JCMM-28-e70079-s002.zip › 7.GSEA analysis/4.MYRF/KEGG_CHEMOKINE_SIGNALING_PATHWAY.html]

Details for gene set KEGG\_CHEMOKINE\_SIGNALING\_PATHWAY[GSEA]

|  || Dataset | MYRF.group.cls#h\_versus\_l |
| Phenotype | group.cls#h\_versus\_l |
| Upregulated in class | l |
| GeneSet | KEGG\_CHEMOKINE\_SIGNALING\_PATHWAY |
| Enrichment Score (ES) | -0.4523516 |
| Normalized Enrichment Score (NES) | -1.5877532 |
| Nominal p-value | 0.010570824 |
| FDR q-value | 0.23623344 |
| FWER p-Value | 0.632 |
Table: GSEA Results Summary

  

Fig 1: Enrichment plot: KEGG\_CHEMOKINE\_SIGNALING\_PATHWAY      
 Profile of the Running ES Score & Positions of GeneSet Members on the Rank Ordered List

  

| SYMBOL | TITLE | RANK IN GENE LIST | RANK METRIC SCORE | RUNNING ES | CORE ENRICHMENT || 1 | PIK3R5 | na | 661 | 0.528 | -0.0029 | No |
| 2 | PF4 | na | 812 | 0.495 | 0.0080 | No |
| 3 | ADCY1 | na | 1675 | 0.385 | -0.0051 | No |
| 4 | ADCY2 | na | 2688 | 0.312 | -0.0248 | No |
| 5 | HCK | na | 3050 | 0.292 | -0.0263 | No |
| 6 | CXCR2 | na | 3923 | 0.257 | -0.0436 | No |
| 7 | CXCL13 | na | 4048 | 0.252 | -0.0395 | No |
| 8 | JAK3 | na | 4412 | 0.241 | -0.0426 | No |
| 9 | ADCY5 | na | 4506 | 0.238 | -0.0379 | No |
| 10 | PPBP | na | 4647 | 0.233 | -0.0348 | No |
| 11 | CXCL6 | na | 4997 | 0.222 | -0.0381 | No |
| 12 | CCL1 | na | 5485 | 0.208 | -0.0458 | No |
| 13 | ITK | na | 6002 | 0.192 | -0.0548 | No |
| 14 | CCR1 | na | 6335 | 0.182 | -0.0588 | No |
| 15 | SOS1 | na | 6706 | 0.172 | -0.0642 | No |
| 16 | CCL17 | na | 6752 | 0.171 | -0.0603 | No |
| 17 | CCR6 | na | 7102 | 0.161 | -0.0654 | No |
| 18 | PIK3R1 | na | 7105 | 0.161 | -0.0605 | No |
| 19 | RASGRP2 | na | 7635 | 0.148 | -0.0713 | No |
| 20 | GNG4 | na | 8161 | 0.135 | -0.0823 | No |
| 21 | BRAF | na | 8198 | 0.133 | -0.0793 | No |
| 22 | CCL15 | na | 8571 | 0.125 | -0.0862 | No |
| 23 | ADCY7 | na | 8605 | 0.125 | -0.0833 | No |
| 24 | ROCK1 | na | 8751 | 0.121 | -0.0838 | No |
| 25 | ARRB1 | na | 9053 | 0.115 | -0.0890 | No |
| 26 | KRAS | na | 9371 | 0.108 | -0.0948 | No |
| 27 | PLCB3 | na | 9511 | 0.106 | -0.0956 | No |
| 28 | BCAR1 | na | 9571 | 0.104 | -0.0941 | No |
| 29 | PLCB2 | na | 9762 | 0.100 | -0.0965 | No |
| 30 | PIK3CG | na | 9794 | 0.100 | -0.0943 | No |
| 31 | ADCY4 | na | 9919 | 0.097 | -0.0949 | No |
| 32 | GNAI3 | na | 9952 | 0.097 | -0.0929 | No |
| 33 | AKT2 | na | 10210 | 0.092 | -0.0975 | No |
| 34 | GNG8 | na | 10364 | 0.089 | -0.0992 | No |
| 35 | XCL1 | na | 10618 | 0.084 | -0.1039 | No |
| 36 | PF4V1 | na | 11198 | 0.075 | -0.1184 | No |
| 37 | PIK3CA | na | 11255 | 0.074 | -0.1177 | No |
| 38 | CCL23 | na | 11318 | 0.073 | -0.1172 | No |
| 39 | CCR5 | na | 11326 | 0.073 | -0.1152 | No |
| 40 | SHC3 | na | 11433 | 0.071 | -0.1161 | No |
| 41 | VAV2 | na | 11454 | 0.071 | -0.1145 | No |
| 42 | PRKX | na | 11663 | 0.068 | -0.1184 | No |
| 43 | VAV3 | na | 11728 | 0.067 | -0.1182 | No |
| 44 | CCR10 | na | 12225 | 0.060 | -0.1307 | No |
| 45 | CCR4 | na | 12407 | 0.058 | -0.1341 | No |
| 46 | PIK3R2 | na | 12535 | 0.056 | -0.1361 | No |
| 47 | STAT1 | na | 12606 | 0.055 | -0.1364 | No |
| 48 | SOS2 | na | 12783 | 0.053 | -0.1399 | No |
| 49 | IKBKB | na | 12994 | 0.050 | -0.1444 | No |
| 50 | CXCR5 | na | 12998 | 0.050 | -0.1430 | No |
| 51 | CCL26 | na | 13127 | 0.049 | -0.1452 | No |
| 52 | ADCY6 | na | 13405 | 0.045 | -0.1518 | No |
| 53 | CSK | na | 13727 | 0.041 | -0.1598 | No |
| 54 | ROCK2 | na | 13790 | 0.040 | -0.1604 | No |
| 55 | PRKCZ | na | 13995 | 0.038 | -0.1651 | No |
| 56 | GNG13 | na | 14122 | 0.036 | -0.1677 | No |
| 57 | CHUK | na | 14348 | 0.034 | -0.1731 | No |
| 58 | MAPK3 | na | 14382 | 0.034 | -0.1730 | No |
| 59 | WAS | na | 14632 | 0.031 | -0.1793 | No |
| 60 | GNAI2 | na | 15256 | 0.025 | -0.1965 | No |
| 61 | STAT5B | na | 15287 | 0.025 | -0.1966 | No |
| 62 | TIAM1 | na | 15716 | 0.021 | -0.2084 | No |
| 63 | CCL11 | na | 15898 | 0.019 | -0.2131 | No |
| 64 | PARD3 | na | 16431 | 0.013 | -0.2281 | No |
| 65 | CXCL5 | na | 16593 | 0.012 | -0.2323 | No |
| 66 | ARRB2 | na | 17178 | 0.007 | -0.2490 | No |
| 67 | PIK3CB | na | 17199 | 0.007 | -0.2494 | No |
| 68 | STAT2 | na | 17245 | 0.006 | -0.2505 | No |
| 69 | RAC2 | na | 17259 | 0.006 | -0.2507 | No |
| 70 | RAP1A | na | 17348 | 0.006 | -0.2531 | No |
| 71 | WASL | na | 17383 | 0.005 | -0.2539 | No |
| 72 | PLCB4 | na | 17554 | 0.004 | -0.2587 | No |
| 73 | XCR1 | na | 17596 | 0.003 | -0.2598 | No |
| 74 | TIAM2 | na | 17643 | 0.003 | -0.2610 | No |
| 75 | ADCY9 | na | 17667 | 0.003 | -0.2616 | No |
| 76 | AKT1 | na | 18101 | -0.001 | -0.2741 | No |
| 77 | GNB2 | na | 18182 | -0.002 | -0.2763 | No |
| 78 | CCL24 | na | 18225 | -0.003 | -0.2775 | No |
| 79 | GNG3 | na | 18263 | -0.003 | -0.2784 | No |
| 80 | CCL18 | na | 18351 | -0.004 | -0.2808 | No |
| 81 | GNG10 | na | 18776 | -0.008 | -0.2929 | No |
| 82 | RHOA | na | 18827 | -0.008 | -0.2941 | No |
| 83 | PTK2B | na | 18851 | -0.009 | -0.2945 | No |
| 84 | RAP1B | na | 18871 | -0.009 | -0.2947 | No |
| 85 | GRK5 | na | 19049 | -0.011 | -0.2995 | No |
| 86 | GSK3B | na | 19466 | -0.016 | -0.3111 | No |
| 87 | GSK3A | na | 19502 | -0.016 | -0.3116 | No |
| 88 | PRKACA | na | 19584 | -0.017 | -0.3134 | No |
| 89 | ADCY8 | na | 20359 | -0.026 | -0.3350 | No |
| 90 | MAPK1 | na | 20406 | -0.027 | -0.3355 | No |
| 91 | PIK3CD | na | 20433 | -0.027 | -0.3355 | No |
| 92 | PIK3R3 | na | 20464 | -0.027 | -0.3355 | No |
| 93 | CXCL14 | na | 20555 | -0.028 | -0.3372 | No |
| 94 | PRKCD | na | 20569 | -0.028 | -0.3367 | No |
| 95 | GNG7 | na | 20692 | -0.030 | -0.3394 | No |
| 96 | GRK6 | na | 21215 | -0.036 | -0.3534 | No |
| 97 | FOXO3 | na | 21594 | -0.041 | -0.3631 | No |
| 98 | IKBKG | na | 21687 | -0.042 | -0.3644 | No |
| 99 | GNG5 | na | 21703 | -0.042 | -0.3636 | No |
| 100 | RAF1 | na | 21720 | -0.042 | -0.3627 | No |
| 101 | HRAS | na | 21856 | -0.044 | -0.3653 | No |
| 102 | RAC1 | na | 21936 | -0.045 | -0.3662 | No |
| 103 | CCL8 | na | 22516 | -0.052 | -0.3813 | No |
| 104 | CCL16 | na | 22560 | -0.053 | -0.3810 | No |
| 105 | CCL27 | na | 22822 | -0.057 | -0.3868 | No |
| 106 | CXCL12 | na | 22943 | -0.059 | -0.3884 | No |
| 107 | GNB5 | na | 23084 | -0.061 | -0.3906 | No |
| 108 | JAK2 | na | 23139 | -0.062 | -0.3903 | No |
| 109 | CDC42 | na | 23297 | -0.064 | -0.3929 | No |
| 110 | CCL19 | na | 23536 | -0.068 | -0.3976 | No |
| 111 | PTK2 | na | 23650 | -0.070 | -0.3988 | No |
| 112 | GNB1 | na | 23971 | -0.075 | -0.4057 | No |
| 113 | GRB2 | na | 24215 | -0.079 | -0.4103 | No |
| 114 | CCR8 | na | 24232 | -0.080 | -0.4083 | No |
| 115 | CXCL16 | na | 24251 | -0.080 | -0.4064 | No |
| 116 | SHC1 | na | 24263 | -0.080 | -0.4043 | No |
| 117 | GNG2 | na | 24281 | -0.080 | -0.4023 | No |
| 118 | CCR3 | na | 24386 | -0.082 | -0.4028 | No |
| 119 | NCF1 | na | 25156 | -0.096 | -0.4221 | No |
| 120 | GNG12 | na | 25293 | -0.099 | -0.4230 | No |
| 121 | CRK | na | 25398 | -0.101 | -0.4229 | No |
| 122 | NFKBIB | na | 25462 | -0.102 | -0.4216 | No |
| 123 | PRKCB | na | 25770 | -0.108 | -0.4271 | No |
| 124 | PAK1 | na | 26325 | -0.120 | -0.4395 | No |
| 125 | STAT3 | na | 26744 | -0.129 | -0.4476 | Yes |
| 126 | CCL28 | na | 26883 | -0.132 | -0.4475 | Yes |
| 127 | ADCY3 | na | 26955 | -0.134 | -0.4455 | Yes |
| 128 | GRK4 | na | 26961 | -0.134 | -0.4415 | Yes |
| 129 | CCL14 | na | 27081 | -0.137 | -0.4407 | Yes |
| 130 | CCR2 | na | 27116 | -0.138 | -0.4375 | Yes |
| 131 | CCL4L2 | na | 27215 | -0.140 | -0.4360 | Yes |
| 132 | CXCR3 | na | 27781 | -0.155 | -0.4476 | Yes |
| 133 | GNB3 | na | 27836 | -0.156 | -0.4444 | Yes |
| 134 | CCL13 | na | 27880 | -0.157 | -0.4408 | Yes |
| 135 | GRK1 | na | 28091 | -0.163 | -0.4418 | Yes |
| 136 | CCR9 | na | 28216 | -0.167 | -0.4403 | Yes |
| 137 | NFKB1 | na | 28241 | -0.168 | -0.4358 | Yes |
| 138 | CXCL11 | na | 28291 | -0.170 | -0.4320 | Yes |
| 139 | PXN | na | 28410 | -0.173 | -0.4301 | Yes |
| 140 | GNAI1 | na | 28414 | -0.173 | -0.4249 | Yes |
| 141 | VAV1 | na | 28682 | -0.181 | -0.4271 | Yes |
| 142 | LYN | na | 28768 | -0.184 | -0.4239 | Yes |
| 143 | CCL2 | na | 28806 | -0.185 | -0.4193 | Yes |
| 144 | CRKL | na | 29092 | -0.194 | -0.4215 | Yes |
| 145 | NRAS | na | 30019 | -0.224 | -0.4415 | Yes |
| 146 | GNGT2 | na | 30023 | -0.224 | -0.4346 | Yes |
| 147 | MAP2K1 | na | 30150 | -0.229 | -0.4312 | Yes |
| 148 | GNGT1 | na | 30222 | -0.231 | -0.4262 | Yes |
| 149 | FGR | na | 30249 | -0.232 | -0.4198 | Yes |
| 150 | CCL7 | na | 30606 | -0.246 | -0.4225 | Yes |
| 151 | CCL21 | na | 30783 | -0.254 | -0.4198 | Yes |
| 152 | PLCB1 | na | 30796 | -0.254 | -0.4124 | Yes |
| 153 | CCL5 | na | 31086 | -0.268 | -0.4125 | Yes |
| 154 | RELA | na | 31135 | -0.271 | -0.4055 | Yes |
| 155 | PRKACG | na | 31385 | -0.285 | -0.4040 | Yes |
| 156 | NFKBIA | na | 31525 | -0.292 | -0.3990 | Yes |
| 157 | GNB4 | na | 31540 | -0.293 | -0.3904 | Yes |
| 158 | CCL22 | na | 32141 | -0.326 | -0.3978 | Yes |
| 159 | CXCR6 | na | 32224 | -0.331 | -0.3900 | Yes |
| 160 | GNG11 | na | 32303 | -0.337 | -0.3819 | Yes |
| 161 | SHC4 | na | 32535 | -0.353 | -0.3777 | Yes |
| 162 | PREX1 | na | 32583 | -0.356 | -0.3681 | Yes |
| 163 | CCR7 | na | 32711 | -0.366 | -0.3605 | Yes |
| 164 | CX3CR1 | na | 33108 | -0.398 | -0.3598 | Yes |
| 165 | CCL3L3 | na | 33123 | -0.399 | -0.3479 | Yes |
| 166 | GRK7 | na | 33181 | -0.405 | -0.3371 | Yes |
| 167 | PRKACB | na | 33438 | -0.435 | -0.3311 | Yes |
| 168 | ELMO1 | na | 33465 | -0.438 | -0.3184 | Yes |
| 169 | CX3CL1 | na | 33908 | -0.505 | -0.3156 | Yes |
| 170 | DOCK2 | na | 33914 | -0.506 | -0.3002 | Yes |
| 171 | CXCL2 | na | 34010 | -0.524 | -0.2868 | Yes |
| 172 | CCL3 | na | 34424 | -0.643 | -0.2790 | Yes |
| 173 | CXCL1 | na | 34529 | -0.693 | -0.2607 | Yes |
| 174 | CXCR4 | na | 34584 | -0.727 | -0.2400 | Yes |
| 175 | CXCR1 | na | 34635 | -0.792 | -0.2170 | Yes |
| 176 | CCL20 | na | 34639 | -0.799 | -0.1926 | Yes |
| 177 | SHC2 | na | 34640 | -0.800 | -0.1679 | Yes |
| 178 | CXCL8 | na | 34657 | -0.838 | -0.1426 | Yes |
| 179 | CCL25 | na | 34662 | -0.842 | -0.1168 | Yes |
| 180 | CXCL9 | na | 34681 | -0.884 | -0.0902 | Yes |
| 181 | CXCL3 | na | 34682 | -0.891 | -0.0628 | Yes |
| 182 | AKT3 | na | 34696 | -0.932 | -0.0345 | Yes |
| 183 | CXCL10 | na | 34719 | -1.151 | 0.0003 | Yes |
Table: GSEA details [plain text format]

  

Fig 2: KEGG\_CHEMOKINE\_SIGNALING\_PATHWAY      
 Blue-Pink O' Gram in the Space of the Analyzed GeneSet

  

Fig 3: KEGG\_CHEMOKINE\_SIGNALING\_PATHWAY: Random ES distribution      
 Gene set null distribution of ES for **KEGG\_CHEMOKINE\_SIGNALING\_PATHWAY**
